# Cytotoxicity and molecular docking analysis of racemolactone I, a new sesquiterpene lactone isolated from *Inula racemosa*

**DOI:** 10.1080/13880209.2021.1946090

**Published:** 2021-07-11

**Authors:** Perwez Alam, Rama Tyagi, Mohammad Abul Farah, Md. Tabish Rehman, Afzal Hussain, Mohamed Fahad AlAjmi, Nasir Ali Siddiqui, Khalid Mashay Al-Anazi, Saima Amin, Mohd. Mujeeb, Showkat R. Mir

**Affiliations:** a Department of Pharmacognosy, College of Pharmacy, King Saud University, Riyadh, Saudi Arabia; b Phyto-Pharmaceutical Research Lab, School of Pharmaceutical Education and Research, Jamia Hamdard, New Delhi, India; c Department of Zoology, College of Science, King Saud University, Riyadh, Saudi Arabia; d Department of Pharmaceutics, School of Pharmaceutical Education and Research, Jamia Hamdard, New Delhi, India; e Department of Pharmacognosy and Phytochemistry, School of Pharmaceutical Education and Research, Jamia Hamdard, New Delhi, India

**Keywords:** Asteraceae, dimeric sesquiterpene, apoptosis, DNA damage detection, *Inula racemosa*

## Abstract

**Context:**

Traditionally, *Inula racemosa* Hook. f. (Asteraceae) has been reported to be effective in cancer treatment which motivated the authors to explore the plant for novel anticancer compounds.

**Objective:**

To isolate and characterize new cytotoxic phytoconstituents from *I. racemosa* roots.

**Materials and methods:**

The column chromatography of *I. racemosa* ethyl acetate extract furnished a novel sesquiterpene lactone whose structure was established by NMR (1D/2D), ES-MS and its cytotoxic properties were assessed on HeLa, MDAMB-231, and A549 cell lines using MTT and LDH (lactate dehydrogenase) assays. Further, morphological changes were analyzed by flow cytometry, mitochondrial membrane potential, AO-EtBr dual staining, and comet assay. Molecular docking and simulation were performed using Glide and Desmond softwares, respectively, to validate the mechanism of action.

**Results:**

The isolated compound was identified as racemolactone I (compound **1**). Amongst the cell lines tested, considerable changes were observed in HeLa cells. Compound **1** (IC_50_ = 0.9 µg/mL) significantly decreased cell viability (82%) concomitantly with high LDH release (76%) at 15 µg/mL. Diverse morphological alterations along with significant increase (9.23%) in apoptotic cells and decrease in viable cells were observed. AO-EtBr dual staining also confirmed the presence of 20% apoptotic cells. A gradual decrease in mitochondrial membrane potential was observed. HeLa cells showed significantly increased comet tail length (48.4 µm), indicating broken DNA strands. *In silico* studies exhibited that compound **1** binds to the active site of Polo-like kinase-1 and forms a stable complex.

**Conclusions:**

Racemolactone I was identified as potential anticancer agent, which can further be confirmed by *in vivo* investigations.

## Introduction

Cancer is the second leading cause of death worldwide. In 2020, there were an estimated 19.3 million new cancer patients and ∼10.0 million deaths occurred worldwide including cervical cancer (approximately 604,000 new patients and 342,000 deaths). Cervical cancer was the fourth leading cause of cancer death in women in 2020 and it was predominantly found in the countries situated in sub-Saharan Africa, Melanesia, South America, and the South-Eastern Asia region (Sung et al. 2021). The major cause of mortality in cancer is metastasis – the recurrence of treatment-resistant cancer cells found locally or in distant organs. In addition, the major reason for treatment failure may be the presence of cancer stem cells in the infected area (Sotillo et al. 2017). The most common approaches to cancer treatment include surgery, chemotherapy, and radiotherapy. However, the dependence of patients on chemotherapy and radiotherapy often results in severe side effects. In addition, certain drugs, such as herceptin, which is used for breast cancer treatment, are expensive and thus out of reach for many patients (Slamon and Pegram 2001). Another drawback of synthetic drugs is that certain cancers develop resistance against them. Synthetic drugs also have numerous side effects such as nephrotoxicity, hepatotoxicity, and bone marrow depression. Therefore, there is an urgent need for identifying and developing novel anticancer drug candidates, requiring the immediate and collective effort of researchers from different scientific fields.

Plants are a rich source of novel, unexplored chemical scaffolds that may contribute to the development of an effective anticancer drug. In fact, more than 50–60% of anticancer drugs currently on the market (such as taxol, vincristine, vinblastine, camptothecin, arglabin, etc.) are derived from natural-products (Gach et al. 2015). *Inula racemosa* Hook. F (Asteraceae) grows widely in the western Himalayas of Xinjiang (China), Afghanistan, Nepal, and almost all parts of India. Traditionally, it has been used since ancient times as a medicine to treat different diseases such as cancer, cardiovascular disorders, dysentery, chronic dyspepsia, and pain (especially between the neck and shoulders) (Firdous et al. 2018). *Inula racemosa* has several pharmacological properties, such as anti-apoptotic (Arumugam and Murugan 2013), cardioprotective (Shirole et al. 2013), antioxidant (Tavares and Seca 2019), and antimicrobial (Lokhande et al. 2007) properties. A large number of secondary metabolites have been isolated from different extracts/fractions of *I. racemosa* using chromatography. These secondary metabolites include eudesmulolide esters (Khan et al. 2014), isoalantolactone, dihydroisoalantolactone, alantodiene, isoalantodiene (Sharma et al. 2016), sesquiterpenoids (Zhang et al. 2012), and sesquiterpene lactones (Bohlmann et al. 1978).

This study isolated a novel sesquiterpene lactone, racemolactone I (compound **1**), for the first time from *I. racemosa* roots and evaluated its cytotoxic potential against cervical cancer (HeLa), breast cancer (MDA MB-231), and lung cancer (A549) cell lines. In addition, we determined the mechanism by which compound **1** exerts its cytotoxicity on HeLa cells by monitoring DNA damage and apoptosis. Finally, the *in vitro* results were further validated by performing molecular docking and molecular dynamics (MD) simulation of compound **1** with Polo-like kinase-1 (PLK-1).

## Materials and methods

### Plant material

We obtained fresh *I. racemosa* roots from Universal Biotech (Gali Chashreen, Farash Khana, Delhi, India) in the month of March 2017. It was identified by Dr. H. B. Singh, Taxonomist, Aimil Pharmaceuticals India Ltd., Delhi, India. A specimen (voucher no. PRL/2017/21) was kept in the Phytochemistry Research Lab, Department of Pharmacognosy, New Delhi, India, for future reference.

### Preparation of methanol extract

The roots were cleaned, washed, and dried in an oven at 45 °C. Next, the dried roots were pulverized to a coarse powder using a grinder, and then ∼2.7 kg of root powder was Soxhlet-extracted with 20 L of methanol for 72 h. The obtained extract was filtered and evaporated under reduced pressure using a rotary evaporator (Buchi, Switzerland) to obtain a dried, brownish, viscous mass of 762 g (yield 28.2%).

### Fractionation and isolation of phytoconstituents

The dried methanol extract was suspended in 1 L of water and fractionated with ethyl acetate (1L thrice). Phytoconstituents were isolated from the obtained from concentrated ethyl acetate fraction using column chromatography (normal-phase medium-pressure liquid chromatography [MPLC]). Preparative separation was achieved by using the Easy Extract Purification System (Buchi, Switzerland) with a 70 × 460 mm plastic-glass column (Büchi, Switzerland) packed with silica gel Si60 (50–60 µm; Merck). Elution with hexane-ethyl acetate (70:30 v/v) resulted in the isolation of racemolactone I (compound **1**), an off-white amorphous powder (yield 0.52%) with retardation factor (*R*_f_) = 0.77 (toluene-chloroform-ethanol, 8:8:2 v/v/v) and melting point 140–142 °C.

### Cytotoxicity assays of compound **1**

#### Cell culture and treatments

We obtained HeLa, MDA MB-231, and A549 cells from the American Type Culture Collection (Rockville, MD, USA) and cultured them in Dulbecco’s modified Eagle’s medium (Sigma-Aldrich, St. Louis, MO, USA) with 10% foetal bovine serum and 1% penicillin/streptomycin at 37 °C in a completely humidified atmosphere with 95% air and 5% CO_2_. Next, we subcultured the exponentially growing cells in 6- or 96-well plates according to experimental requirements. Cell viability was determined using the trypan blue test, and the cells were counted using a Bio Rad TC20 automated cell counter (Bio-Rad Laboratories, Hercules, CA, USA) and diluted in a medium at a density of 1 × 10^5^ cells/mL to be used for further experiments. We also prepared a stock solution of compound **1** in dimethyl sulfoxide (DMSO) (w/v) and diluted it in a cell culture medium to obtain the desired concentrations for cell treatment.

#### Cytotoxicity assay

We analyzed the cytotoxicity of compound **1** with 3-(4,5-dimethylthiazol-2-yl)-2,5-diphenyltetrazolium bromide (MTT) assay using a CellTitre 96^®^ nonradioactive cell proliferation assay kit (Promega, Madison, WI, USA) according to the manufacturer’s instructions. Briefly, HeLa, MDA MB-231, and A549 cells were grown overnight in 96-well flat-bottom cell culture plates at a density of 1 × 10^4^ cells/well and then exposed to six different concentrations of compound **1** (15, 10, 5, 1, 0.75, and 0.5 µg/mL) for 24 h. Untreated cells were used as a negative control. After treatment, we added 15 µL of MTT reagent, provided in the kit, to each well and further incubated the cells for 3 h at 37 °C. Finally, we removed the medium with MTT reagent, added 200 µL of solubilization solution to each well, and further incubated the cells for 30 min with occasional vortexing. We measured the optical density of each well at a wavelength of 550 nm using a Synergy microplate reader (BioTek, Winooski, VA, USA). Results were generated from three independent experiments, and each experiment was performed in triplicate. In addition, we determined the percentage of cytotoxicity compared to untreated cells in order to calculate the median inhibitory concentration (IC_50_), the concentration at which 50% cell proliferation is inhibited.

#### Lactate dehydrogenase cytotoxicity assay

We assessed the plasma membrane integrity of HeLa cells by estimating the amount of lactate dehydrogenase (LDH) present in the culture medium. Briefly, we cultured HeLa cells in 96-well plates at a density of 1 × 10^4^ cells/well and then exposed them to six different concentrations of compound **1** (15, 10, 5, 1, 0.75, and 0.5 µg/mL) for 24 h. After treatment, we performed LDH cytotoxicity assay according to the manufacturer’s instructions (Abcam). Briefly, the culture plates were centrifuged at 600 × *g* for 10 min to precipitate the cells, 50 μL of clear cell culture supernatant was transferred from each well to a 96-well plate, and 100 μL of freshly prepared LDH reaction mixture was added to each well. After 30 min incubation at room temperature in the dark, absorbance was measured at a wavelength of 450 nm using a Synergy microplate reader (BioTek, Winooski, VA, USA). The LDH content was expressed as a percentage compared to control cells, which was considered 100%.

### Morphological changes in HeLa cells

HeLa cells were seeded in a 6-well plate at a density of 1 × 10^5^ cells/well and allowed to grow overnight. Morphological changes were observed to determine alterations induced by two sublethal concentrations (0.5 and 0.75 µg/mL) of compound **1**. After 24 h incubation, the cells were washed with phosphate-buffered saline (PBS; pH 7.4) and observed under a phase-contrast inverted microscope equipped with an Olympus IX51 charge-coupled divide (CCD) camera (Olympus, Tokyo, Japan) at 100× magnification.

### Annexin V – FITC apoptosis assay by flow cytometry

We measured apoptosis of HeLa cells using the annexin V–propidium iodide (PI) double-staining method with the annexin V–fluorescein isothiocyanate (FITC) apoptosis detection kit (BD Biosciences, San Diego, CA, USA). Briefly, 1 × 10^5^ cells/mL were grown overnight in 6-well plates and exposed to 0.5 and 0.75 µg/mL of compound **1** for 24 h. Next, the cells were washed with cold PBS, trypsinized, and centrifuged at 1000 rpm, and the cell pellet was rewashed with PBS and resuspended in 100 µL of 1× binding buffer (1 × 10^6^ cells/mL). We added 5 µL each of annexin V–FITC and PI to the cell suspension, gently vortexed the cells, and then incubated them for 20 min at room temperature (25 °C) in the dark. Subsequently, we diluted the samples by adding 400 µL 1× binding buffer. Annexin-V/PI fluorescence was analyzed for each sample using a BD FACS Calibur flow cytometer (BD Biosciences). We acquired 10,000 events for each sample and analyzed the data using Cell Quest Pro software (BD Biosciences).

### Apoptotic induction analysis by acridine orange-ethidium bromide dual staining

We used the acridine orange-ethidium bromide (AO-EB) dual-staining method to differentiate between condensed apoptotic or necrotic nuclei and normal cells. Briefly, HeLa cells were seeded on a coverslip-loaded 6-well plate at a density of 1 × 10^5^ cells/well and allowed to grow overnight. Then, the cells were exposed to 0.5 and 0.75 µg/mL of compound **1** for 24 h, washed twice with PBS to remove the remaining medium, and stained with equal volumes of AO and EB (20 μg/mL in PBS). Finally, the stained cells were washed with PBS and mounted onto a microscope slide in mounting medium, and images were obtained using appropriate filter settings in an Olympus BX41 compound microscope (Olympus) fitted with a fluorescence attachment and CCD camera. We also quantified apoptotic and necrotic cells on the basis of the uptake of AO and EB in more than 300 cells. The criteria for identification were as follows: a green intact nucleus and viable cells; dense green areas of chromatin condensation in the nucleus and apoptosis; and an orange intact nucleus and necrosis.

### Changes in mitochondrial membrane potential (δψm)

To detect the early stages of apoptosis, we measured the mitochondrial membrane potential (MMP) in treated and control HeLa cells by staining with a fluorescent probe, rhodamine 123. Briefly, HeLa cells were seeded on a coverslip-loaded 6-well plate at a density of 1 × 10^5^ cells/well, allowed to adhere for 24 h, then treated with 0.5 and 0.75 µg/mL of compound **1**, and further incubated for 24 h. Next, the cells were washed with PBS (pH 7.4), fixed with ice-cold 70% ethanol, and incubated with 5 µg/mL of rhodamine 123 for 30 min at 37 °C. Finally, the cells were again washed with PBS and mounted onto a microscope slide in mounting medium, and images were obtained using appropriate filter settings in an Olympus BX41 compound microscope (Olympus) at 400× magnification.

### Detection of DNA damage by comet assay

To detect the DNA damage in HeLa cells, we performed the alkaline comet assay, as previously described (Farah et al. 2016) based on the original work of Singh et al. (1988) with additional modifications. Briefly, 1 × 10^5^ cells/well were seeded in a 5 mL culture flask and grown overnight. Next, the cells were then treated with 0.5 and 0.75 µg/mL of compound **1** for 24 h, trypsinized, and resuspended in cold PBS. About 15 µL of cell suspension (∼10,000 cells) was mixed with 100 µL of 0.75% low-melting agarose at 37 °C and spread on frosted glass slides precleaned and precoated with a 150 µL layer of 1.5% normal melting agarose. After solidification, the slides were covered with a third layer of 100 µL of 0.75% low-melting agarose, covered with a coverslip, and allowed to solidify on ice for 20 min. Next, the coverslips were removed gently, and the slides were immersed in freshly prepared ice-cold lysis solution (10 mM Tris [pH 10], 2.5 M NaCl, and 100 mM disodium ethylenediaminetetraacetic acid [Na_2_EDTA] with 10% DMSO and 1% Triton X-100 added just before use) at 4 °C for 2 h. After lysis treatment, the slides were kept in an alkaline electrophoresis buffer (300 mM NaOH, 1 mM EDTA; pH > 13) for 20 min in a horizontal gel electrophoresis unit for DNA unwinding and conversion of alkali-labile sites to single-strand breaks. Next, electrophoresis was performed for 20 min by applying an electric field of 25 V and adjusting the current to 300 mA. The slides were neutralized gently with 0.4 M Tris buffer at pH 7.5, and each slide was stained with 75 µL of 20 µg/mL of ethidium bromide solution for 3 min. The slides were analyzed using an Olympus BX41 fluorescence microscope with appropriate filter settings (excitation wavelength 515–560 nm and emission wavelength 590 nm) (Olympus), and 200 cells from each category were randomly selected and subjected to image analysis using Comet Assay IV software (Perceptive Instruments, Suffolk, UK).

### Molecular docking and simulation studies of compound **1**

We evaluated the mode of binding and interaction between PLK-1 and compound **1** by performing molecular docking and MD simulation, as previously described (AlAjmi et al. 2018). Briefly, the 3D structure of compound **1** was drawn in a 2D sketcher (Schrodinger, LLC, NY, USA). Before molecular docking, compound **1** was optimized in LigPrep (Schrodinger) by defining its ionization states at pH 7.0 ± 2.0 using Epik (Schrodinger). We generated a maximum of 32 different stereoisomers of compound **1**, and the energy was minimized using optimized potentials for a liquid simulation 3e (OPLS3e) force field (Schrodinger). We also downloaded the 3D coordinates of the PLK-1 kinase domain (Id: 2OWB) from the Protein Data Bank (Kothe et al. 2007). A nonhydrolyzable adenosine triphosphate (ATP) analog (PHA-680626) was bound to the X-ray crystal of PLK-1. We prepared the PLK-1 structure for molecular docking and simulation by adding hydrogen atoms, assigning bond orders, and deleting any other nonprotein atoms/molecules, including non-essential water molecules, using the protein preparation wizard in Maestro (Schrodinger). In addition, we added missing loops/side chains using Prime (Schrodinger), optimized a hydrogen bond network, and minimized the energy of the whole system using the OPLS3e force field. Finally, a 27 × 27 × 27 Å grid box located at −0.1 × 23.4 × 68.0 Å was generated by picking the bound ligand (PHA-680626) as the grid box centroid using a receptor-grid generation module (Schrodinger), and molecular docking was performed using the extra-precision (XP) mode of Glide (Schrodinger). The binding affinity (*K_d_*) of compound **1** towards PLK-1 was calculated as follows (Rehman et al. 2014; Al-Shabib et al. 2018):

$$\Delta G=-RT\;\text{ln}\;K_{d}$$

where Δ*G* is the binding energy, *R* is the universal gas constant, and *T* is the temperature.

Next, we performed an MD simulation for 50 ns using Desmond (Schrodinger), as previously described. Briefly, we created an orthorhombic box around the compound **1**–PLK-1 complex such that the boundaries of the box were at least 10 Å away from the complex. Next, the simulation box was solvated with a TIP3P explicit water model and neutralized by adding counter-ions. The physiological conditions were mimicked by adding 0.15 M NaCl, and the entire system was minimized with 1000 iterations with a convergence criterion of 1 kcal/mol/Å using the OPLS3e force field. The simulation was performed for 50 ns using the NPT ensemble at 300 K and 1.013 bars, keeping other settings at default values. We used the Noose–Hoover chain thermostat and the Matrtyna–Tobias Klein barostate to keep the temperature and pressure constant. A time step of 2 fs was maintained during the simulation, while at 10 ps, energies and structures were saved.

### Statistical analysis

All experiments were performed as three independent replicates, and values were presented as the mean ± standard error of the mean. Data were statistically analyzed using Student’s *t*-test for comparison between the means, and *p* < 0.05 was considered statistically significant.

## Results

### Isolation of compound **1**

**Figure 1.**
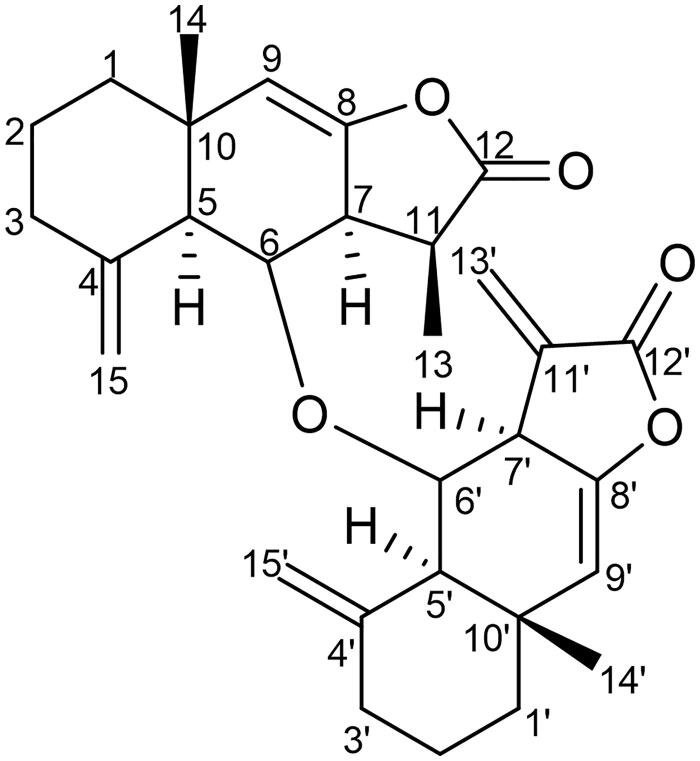
Chemical structure of compound **1** (Racemolactone I).

Elution of the ethyl acetate fraction with a mixture of hexane-ethyl acetate in a 70:30 (v/v) ratio resulted in the isolation of an off-white amorphous powder (compound **1**; Figure 1) with UV *λ*_max_ (MeOH): 225, 268 nm; Fourier transform infra-red (FTIR) (KBr) *ν*_max_: 2935, 2843, 1753, 1645, 1454, 1369, 1259, 1151, 952, 813, and 624 cm^−1^; ^1^H and ^13^C nuclear magnetic resonance (NMR) (CDCl_3_): see Table 1; and positive electrospray mass spectrometry (ES-MS) *m*/*z* (rel. int.): 476 [M]^+^ C_30_H_36_O_5_ (N.O.), 245 [C_15_H_17_O_3_]^+^ (12), and 231 [C_15_H1_9_O_2_]^+^ (48).

**Table 1.** ^1^H and ^13^C NMR spectroscopic data (*δ* ppm) of compound **1**.

Position	^1^H NMR	^13^C NMR	DEPT-135
1	1.37	41.8	CH_2_
2	1.34	21.2	CH_2_
3	1.97	36.7	CH_2_
4		142.2	C
5	1.83 days (8.0)	46.2	CH
6	4.48 m	76.8	CH
7	2.78 m	40.5	CH
8		148.9	C
9	5.35 s	121.2	CH
10		34.2	C
11	2.32 days (8.0)	40.4	CH
12		179.4	C
13	1.32 days (9.0)	9.3	CH_3_
14	0.85 s	18.2	CH_3_
15	4.74 s, 4.41 s	106.4	CH_2_
1′	1.37	42.1	CH_2_
2′	1.34	22.6	CH_2_
3′	1.97	27.4	CH_2_
4′		141.2	C
5′	1.82 days (8.5)	46.5	CH
6′	4.46 m	77.8	CH
7′	2.89 m	41.3	CH
8′		148.9	C
9′	5.35 s	121.2	CH
10′		34.8	C
11′		149.4	C
12′		170.6	C
13′	6.10 s, 5.56 s	120.0	CH_2_
14′	0.82 s	18.1	CH_3_
15′	4.74 s, 4.41 s	106.6	CH_2_

Coupling constants J in Hertz are provided in parentheses.

Overlapped signals are included without designated multiplicities.

The FTIR spectrum of compound **1** displayed characteristic absorption bands for γ-lactone (1753 cm^−1^) and unsaturation (1645 and 1454 cm^−1^). On the basis of ^13^C/DEPT and mass spectra, the molecular weight of compound **1** was determined to be 476, corresponding to the molecular formula C_30_H_36_O_5_ of a dimeric sesquiterpene derivative. The formula indicated the presence of 13 double-bond equivalents, 6 of which were adjusted in 2 tricyclic frameworks, 5 in vinylic linkages, and the remaining 2 in carbonyl functionalities. The HR-ES-MS spectrum of compound **1** displayed two major fragment ion peaks at *m*/*z* =245 [C_15_H_17_O_3_]^+^ and 231 [C_15_H1_9_O_2_]^+^ arising because of fission of the C–O–C linkage between two different sesquiterpenic units (asymmetric dimer). The ^1^H NMR spectrum of compound **1** displayed two one-proton broad signals at *δ* 6.10 and 5.56 ascribed correspondingly to H-13'a and H-13'b protons, respectively, of exocyclic vinylic linkage. In addition, a pair of singlets at *δ* 4.74 and 4.41, each integrating for two protons, were assigned to H-15a/H-15'a and H-15b/H-15'b protons, respectively, of the other two exocyclic vinylic linkages. The corresponding signals for vinylic carbons resonated at *δ* 149.4 (C-11′) and 120.0 (C-13′); 142.2 (C-4) and 106.4 (C-15); and 141.2 (C-4′) and 106.6 (C-15′) in the ^13^C NMR spectrum. The signals for two carbonyl carbons at *δ* 179.4 (C-12) and 170.6 (C-12′) supported the presence of two lactone units. A two-proton singlet at *δ* 5.35 was ascribed to H-9 and H-9′ vinylic protons. Two one-proton multiplets at *δ* 4.48 and 4.46 were assigned to oxygenated methine protons (H-6 and 6′). The corresponding signals for the carbons linking two sesquiterpenic residues resonated at *δ* 76.8 (C-6) and 77.8 (C-6′) in the ^13^CNMR spectrum. Two three-proton singlets at *δ* 0.85 (s, H_3_-14) and *δ* 0.82 (s, H_3_-14′), and a three-proton doublet at *δ* 1.32 (*J* = 9.0 Hz, H_3_-13) in ^1^H NMR spectra were attributed to methyl protons. Extensive examination of the 1D and 2D NMR spectra revealed the presence of 30 carbon resonances comprising 9 quaternary carbons, 9 methine carbons, 9 methylene, and 3 methyl carbons. The HMBC experiment showed a significant correlation between H-6 and C-6′; H_3_-13 and C-12; H-7 and C-12; and H-7′ and C-12′. The NMR data of compound **1** were compared to bialantolactone (Jiang et al. 2011). Therefore, compound **1** was found to be 6-(eudesma-4(15), 8, 11(13)-trien-12, 8-olide-6-oxy) eudesma-4(15), 8-dien-12, 8-olide, a new heteromeric sesquiterpene lactone dimer from *I. racemosa* and was designated as racemolactone I.

### Cytotoxicity of compound **1**

#### Cytotoxicity assay

**Figure 2.**
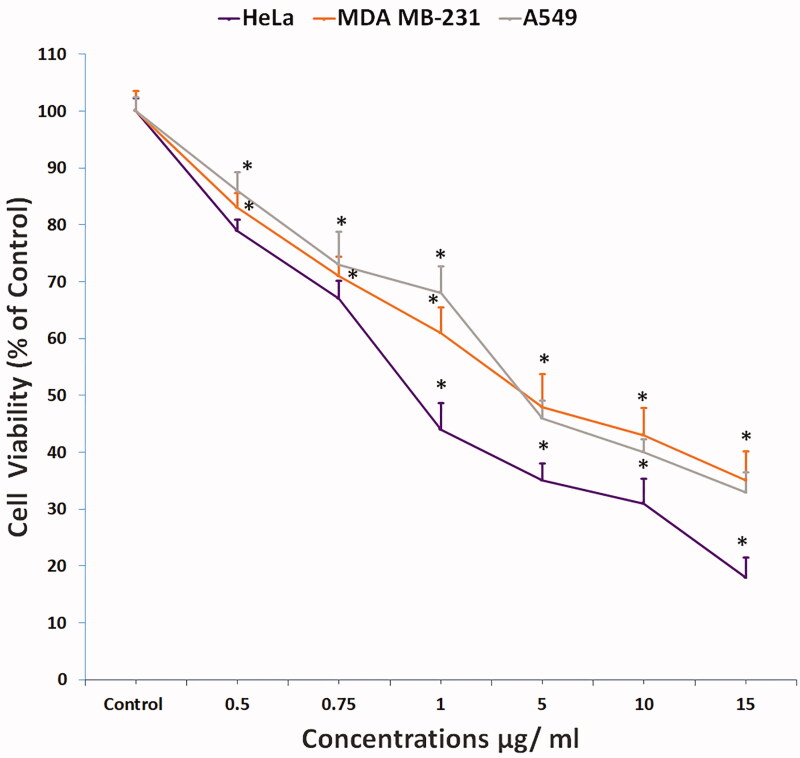
Cytotoxicity of compound **1** on HeLa cells treated in concentrations as indicated for 24 h and analysed by MTT assay (All data are expressed as mean ± SE from three independent experiments). *Significant *p* < 0.05 compared with corresponding controls.

We used three human cancer cell lines, that is, cervical cancer (HeLa), breast cancer (MDA MB-231), and lung cancer (A549), for preliminary screening of compound **1**, and its cytotoxicity was evaluated by standard MTT assay. Cell viability significantly decreased (*p* < 0.05) in all three cell lines in a concentration-dependent manner (Figure 2), and cell proliferation in HeLa, MDA MB-231, and A549 cells was inhibited to 82%, 65%, and 67%, respectively, at the highest compound **1** concentration of 15 µg/mL. IC_50_ at 24 h post-treatment in HeLa, MDA MB-231, and A549 cells was 0.9, 4, and 3.8 µg/mL, respectively. These data showed that compound **1** has higher cytotoxicity in HeLa cells compared to MDA MB-231 and A549 cells. Therefore, we selected HeLa cells for subsequent experiments for apoptosis and DNA damage detection with two sub-lethal concentrations of compound **1**.

#### LDH release analysis

**Figure 3.**
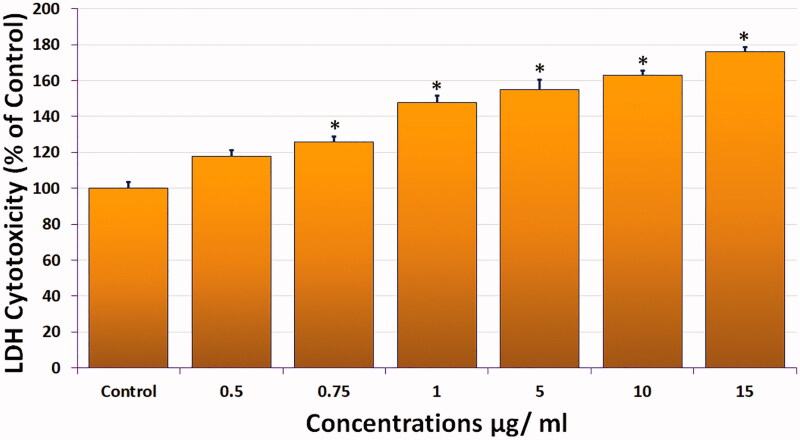
Cytotoxicity of compound **1** measured by LDH release from HeLa cells exposed as indicated for 24 h. (All data are expressed as mean ± SE from three independent experiments). *Significant *p* < 0.05 compared with corresponding controls.

We measured the LDH released into the culture medium in compound **1**-treated HeLa cells as a marker of cell membrane integrity (Figure 3). Compound **1** caused a concentration-dependent release of LDH (18% and 76%) at the lowest (0.5 µg/mL) and highest (15 µg/mL) concentrations, respectively, indicating disruption of the cell membrane structure. LDH release was significant (*p* < 0.05) at all concentrations used except the lowest dose.

#### Morphological change analysis

**Figure 4.**
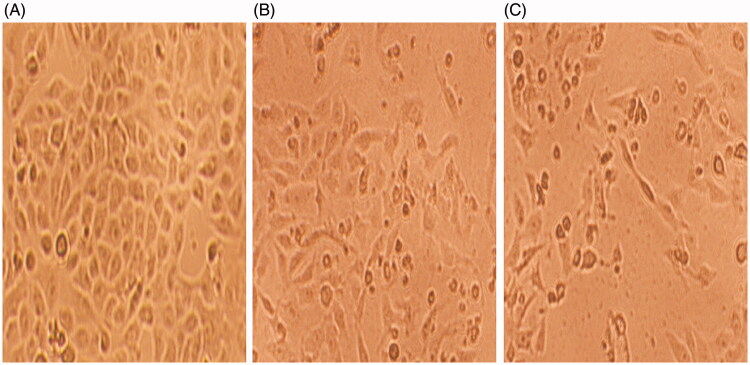
Phase contrast inverted microscope images of HeLa cells showing changes in the morphology of compound **1** treated cells. (A) Untreated control (B) 0.5 µg/mL (C) 0.75 µg/mL. Magnification: 100×.

Figure 4 shows representative images of diverse morphological alterations in HeLa cells post-treatment with compound **1** for 24 h. Control cells had a normal shape, were attached to the surface, and reached approximately 95–100% confluence (Figure 4(A)). We observed morphological variations, such as loss of cell attachment, round or swelled cells, and elongated cells, in compound **1**-treated HeLa cells. The cells underwent growth inhibition because of which the cell density decreased and many cells appeared to be floating in the culture medium in compound **1**-treated HeLa cells (Figure 4(B,C)).

#### Apoptosis analysis by flow cytometry

**Figure 5.**
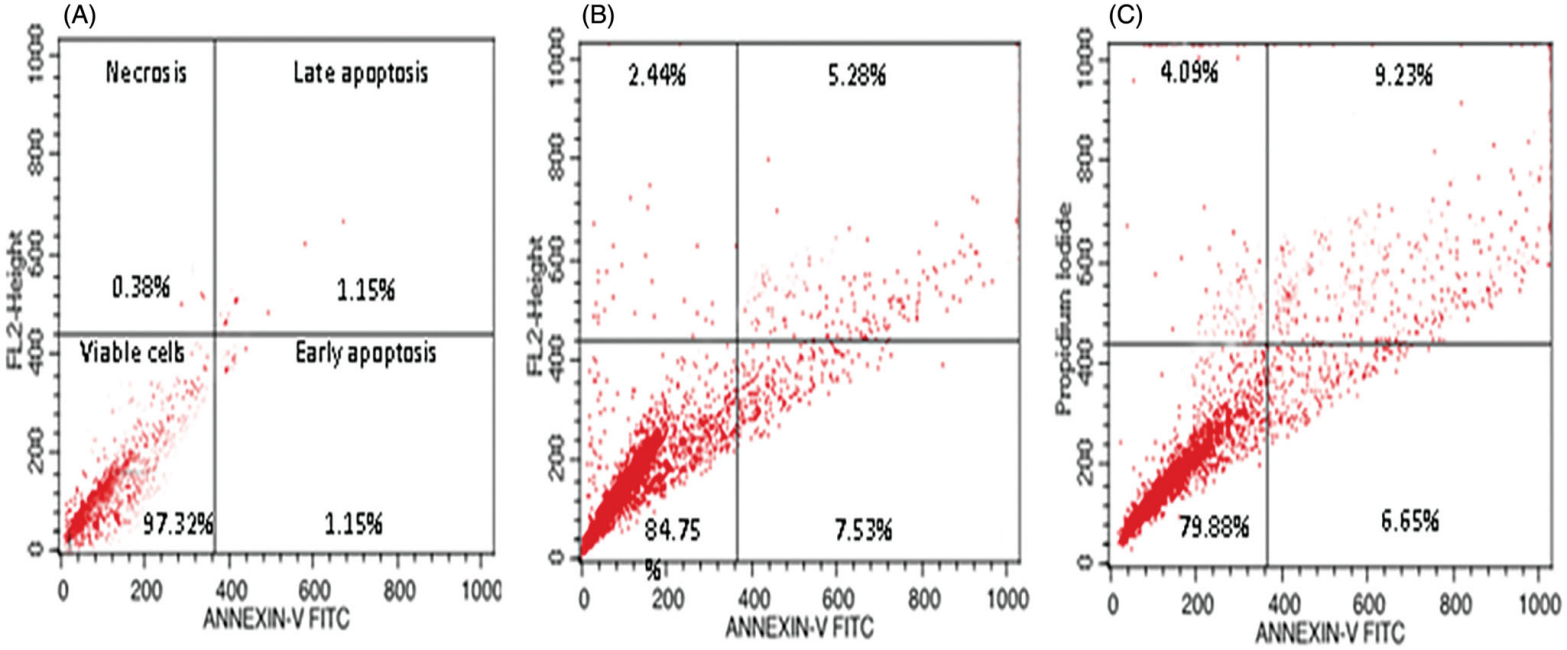
Apoptosis detection by annexin V-FITC/PI staining of HeLa cells analyzed by flow cytometry showing percentage of viable cells, early apoptosis, late apoptosis and necrotic cells. (A) Control (B) 0.5 µg/mL, and (C) 0.75 µg/mL.

We determined the percentages of apoptotic and necrotic cells with annexin V-FITC and PI double staining via flow cytometry; representative results are presented in Figure 5. We designated the positioning of quadrants on dot plots and identified living cells (annexin V−/PI−), early apoptotic cells (annexin V+/PI−), late apoptotic cells (annexin V+/PI+), and necrotic cells (annexin V−/PI+). Compound **1** treatment of HeLa cells decreased the population of viable cells and increased the percentage of apoptotic cells, while in control cells, we observed a negligible percentage of apoptotic cells (Figure 5(A)). The percentage of early apoptotic cells was 7.58% in 0.5 µg/mL and 6.65% in 0.75 µg/mL of compound **1** (Figure 5(B,C)), while the percentage of late apoptotic cells reached to 5.28% and 9.23% in 0.5 and 0.75 µg/mL of compound **1**, respectively. We also observed a small percentage of necrotic cells in compound **1**-treated HeLa cells.

#### Apoptotic induction analysis by acridine orange-ethidium bromide dual staining

**Figure 6.**
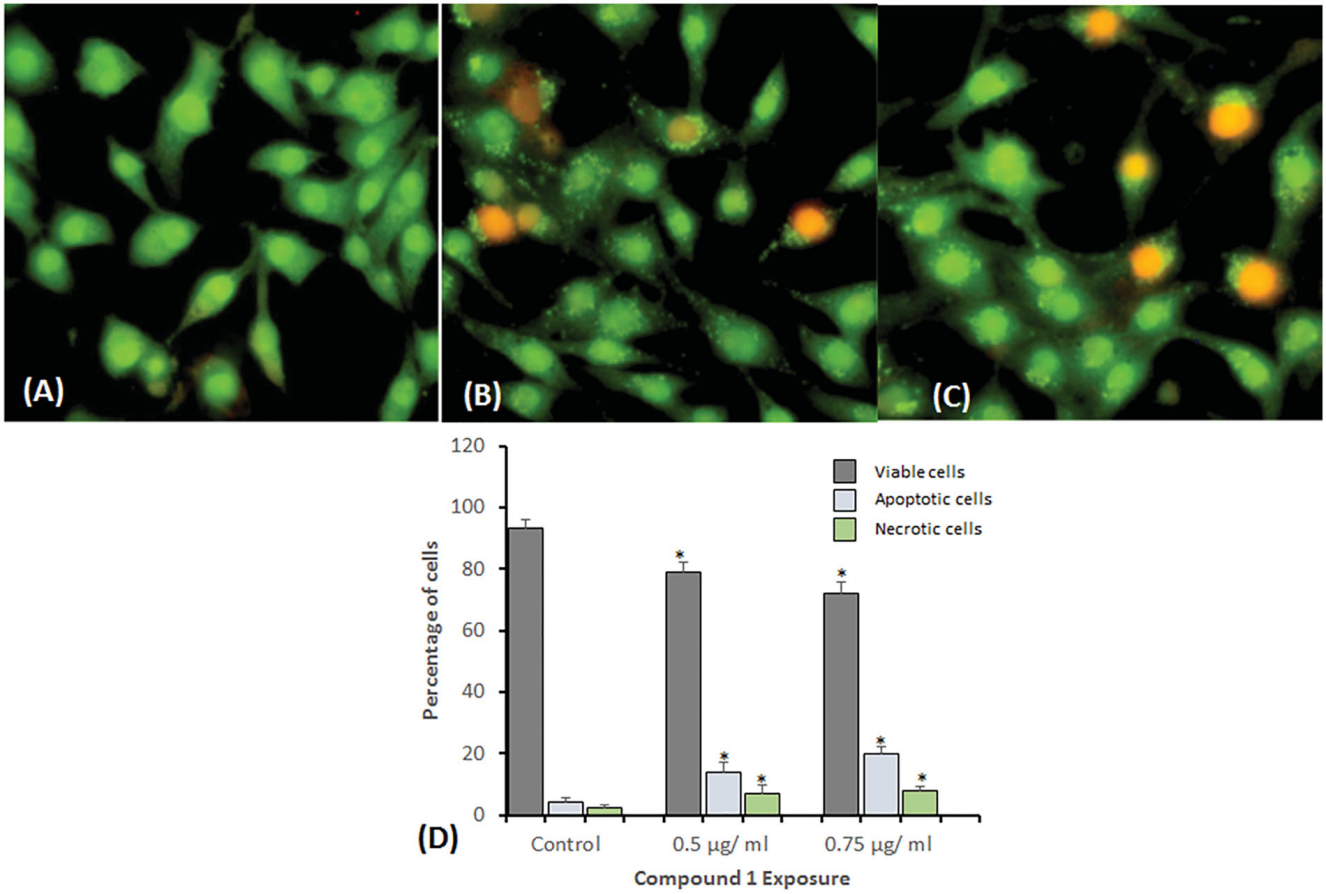
Dual staining with acridine orange and ethidium bromide for the analysis of apoptotic morphological changes in HeLa cells induced by compound **1** and observed under fluorescence microscopy. (A) Control (B) 0.5 µg/mL, and (C) 0.75 µg/mL. Magnification: 200×. (D) Quantification of apoptotic and necrotic cells based on the uptake of acridine orange and ethidium bromide in more than 300 cells. All data are expressed as mean ± SE. *Significant *p* < 0.05 compared with corresponding controls.

To investigate the underlying reason for the increase in cell death, HeLa cells were exposed to two sublethal concentrations (0.5 and 0.75 µg/mL) of compound **1** for 24 h and stained with (AO-EB). We observed ∼92.2% viable control cells, with evenly distributed AO stain (green fluorescence), normal nuclear morphology, and no red fluorescence (Figure 6(A)). In contrast, cell viability significantly decreased in compound **1**-treated HeLa cells. However, the percentage of apoptotic cells significantly increased (*p* < 0.05) at all concentrations, as observed by red cells (Figure 6(B,C)). We quantified 300 cells in each group for scoring apoptotic and necrotic cells and found that compound **1** treatment induces the highest percentage of apoptotic (20%) and necrotic (8%) cells at the highest concentration of 0.75 µg/mL (Figure 6(D)).

#### Mitochondrial membrane potential (δψm) analysis

**Figure 7.**
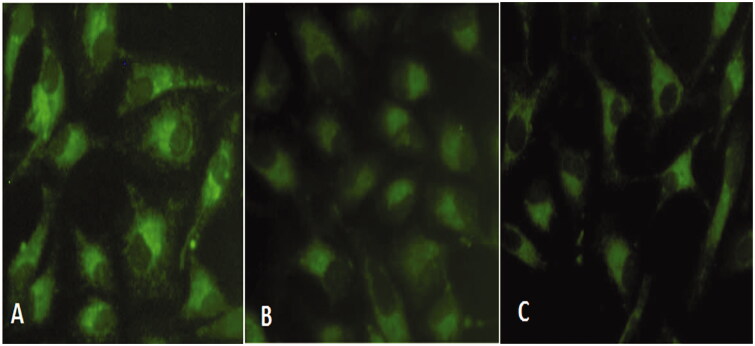
Mitochondrial membrane potential (MMP) analysis in HeLa cells exposed to compound 1 for 24 h and stained with Rhodamine 123. (A) Control (B) 0.5 µg/mL, and (C) 0.75 µg/mL.

The MMP in HeLa cells was measured using the fluorescent probe rhodamine 123 at 24 h post-treatment. Fluorescence microscopic observation of control cells indicated a high MMP, as evidenced by the high-intensity green fluorescence (Figure 7(A)). However, compound **1** treatment significantly decreased the green fluorescence intensity with an increase in concentration compared to control cells (Figure 7(B,C)). This gradual decrease in green fluorescence indicated mitochondrial membrane depolarization, which is an early sign of apoptosis.

#### DNA damage analysis by comet assay

**Figure 8.**
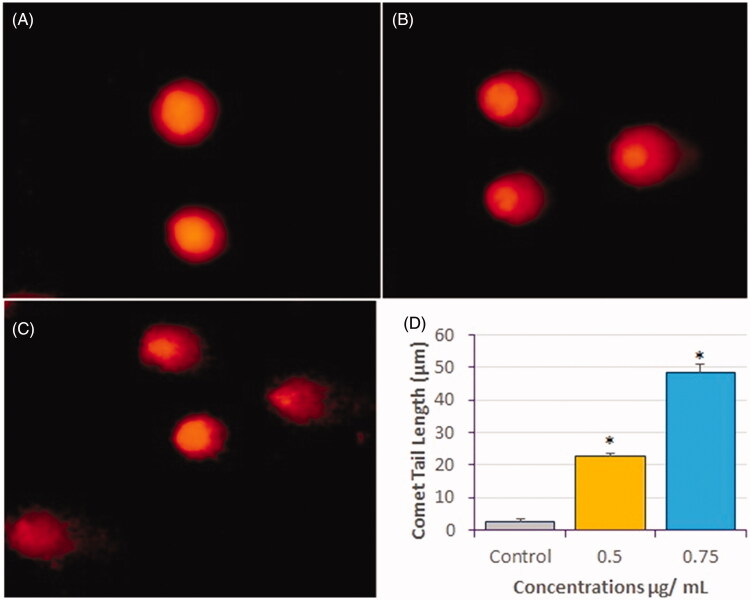
DNA-damage analysis in HeLa cells by comet assay after treatment with compound 1 for 24 h. Representative images of comets are shown. (A) Control (B) 0.5 µg/mL, and (C) 0.75 µg/mL, and (D) Measurement of comet tail length presented as mean ± SE from three independent experiments. *Significant, *p* < 0.05.

Representative images of comets obtained as a result of the genotoxic effect of compound **1** on HeLa cells after 24 h treatment are presented in Figure 8(A–C). The comet tail length significantly increased (*p* < 0.05) in compound **1**-treated HeLa cells compared to control cells. The average comet tail length was ∼2.65 µm in control cells and 22.5 and 48.4 µm in compound **1**-treated HeLa cells at 0.5 and 0.75 µg/mL of compound **1**, respectively (Figure 8(D)). We also observed an increased DNA tail length post-treatment, indicating DNA strand breaks as well as alkali-labile sites. These results confirmed the ability of compound **1** to induce DNA damage at the single-cell level, which may lead to apoptosis.

### Molecular docking and simulation of compound **1**

**Figure 9.**
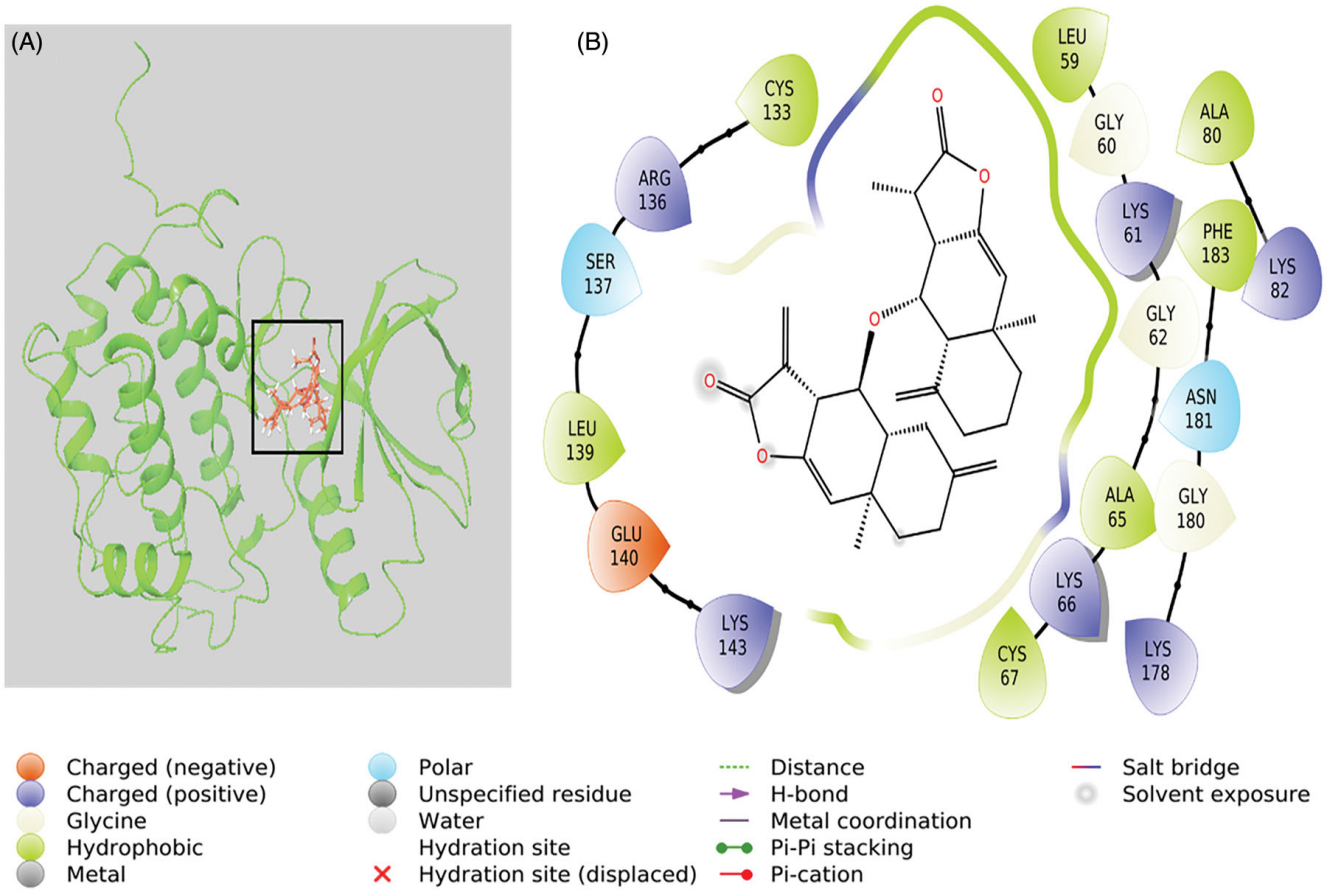
Interaction between compound-1 and PLK-1 kinase domain. (A) Molecular docking of compound 1 to the catalytic site of PLK-1, and (B) Amino acid residues of PLK-1 interacting with compound **1**.

**Figure 10.**
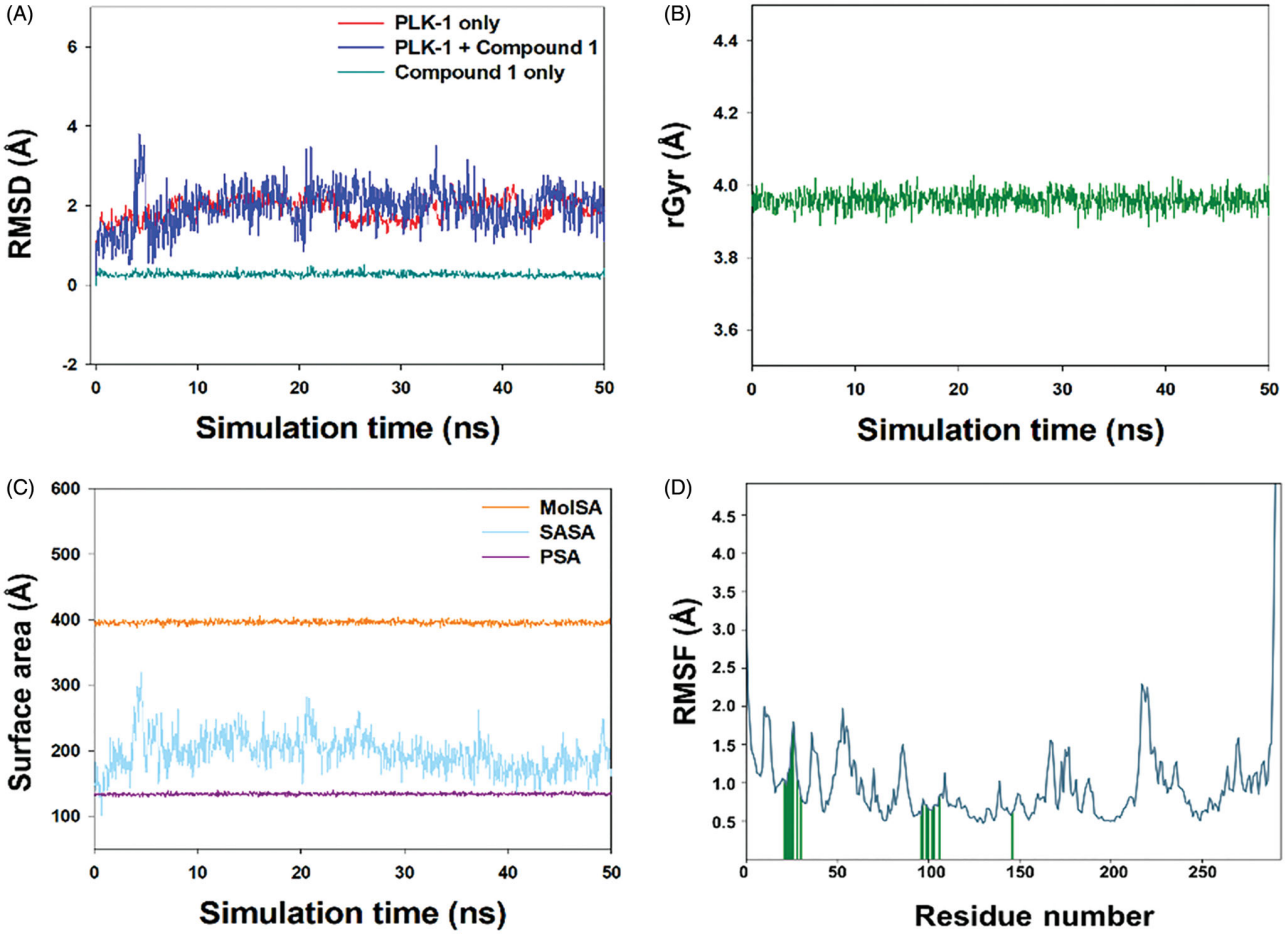
Molecular dynamics simulation of compound **1** and PLK-1. (A) RMSD plot of compound **1**-PLK-1 complex as function of simulation time, (B) variation in the radius of gyration (rGyr) of compound **1**, (C) Variation in molecular surface area (MolSA), solvent accessible surface area (SASA), and polar surface area (PSA) of compound **1** and PLK-1 complex as a function of simulation time, and (D) RMSF variations of PLK-1 interacting with compound **1**.

We gained insight into the mechanism of inhibition of cell proliferation in HepG2 cells by compound **1** treatment by performing molecular docking and simulation studies using the PLK-1 kinase domain. Compound **1** bound to the PLK-1 active site and formed a stable complex (Figure 9(A)). It underwent various van der Waals interactions with Leu59, Gly60, Lys61, Gly62, Ala65, Lys66, Cys67, Ala80, Lys82, Cys133, Arg136, Ser137, Leu139, Glu140, Lys143, Lys178, Gly180, Asn181, and Phe183 (Figure 9(B)). The Gibb’s free energy and binding affinity of the compound **1**–PLK-1 interaction were estimated to be −6.125 kcal/mol and 3.11 × 10^4^ M^−1^, respectively. In addition, stability of the complex between compound **1** and the PLK-1 kinase domain was achieved by performing an MD simulation mimicking physiological conditions (Figure 10) for 50 ns using the initial conformations of compound **1** bound to the PLK-1 kinase domain, as predicted by XP molecular docking. Figure 10 shows the root-mean-square deviation (RMSD) of PLK-1 and compound **1** alone or in a complex with regard to the initial original frame. The RMSD of PLK-1 showed a little deviation for the first 10 ns and then stabilized to 1.9 Å. However, the RMSD of compound **1** was constant at 0.3 Å for the entire simulation time. In addition, the RMSD of compound **1** bound to the PLK-1 catalytic site showed initial fluctuations higher than the upper limit of 2.5 Å and later stabilized to ∼2.0 Å in the 10–50 ns of simulation time (Figure 10(A)). These results indicated that the initial fluctuations in the RMSDs of PLK-1 and compound **1** are due to the addition of a big compound into the PLK-1 catalytic cavity.

We also computed the radius of gyration (rGyr) of compound **1** to determine the conformational stability of compound **1** as a function of simulation time. The rGyr of compound **1** remained constant at ∼3.9 Å, indicating that compound **1** does not undergo a significant conformational change (Figure 10(B)). We also evaluated the formation of a stable compound **1**–PLK-1 complex by monitoring any change in the molecular surface area (MolSA), polar surface area (PSA), and solvent-accessible surface area (SASA) as a function of simulation time. The MolSA, PSA, and SASA of the compound **1**–PLK-1 complex remained constant within limits (Figure 10(C)).

In addition, we calculated RMSFs along the PLK-1 chain to monitor any local conformational changes due to the binding of compound **1** (Figure 10(D)). The fluctuations of PLK-1 side chains were within the prescribed limit, as previously described (AlAjmi et al. 2018). The vertical lines (green in color) on the *x*-axis indicate the position of the amino acid residue in PLK-1, which was in contact with compound **1**. These results showed that compound **1** forms a stable complex with the PLK-1 kinase domain.

## Discussion

Cancer is a principal cause of mortality worldwide and thus poses a serious socioeconomic burden. Plant-derived natural products represent a vast source of a structurally varied pool of compounds that are used to treat different diseases, including various types of cancer. In this study, we isolated a novel sesquiterpene lactone, racemolactone I, for the first time from the roots of *I. racemosa* and evaluated it for cytotoxicity. Principally, sesquiterpene lactones show anticancer and anti-inflammatory activity (Chadwick et al. 2013), in addition to several other biological activities, and have been used in the treatment of diarrhoea, influenza, burns, neurodegeneration, microbial and fungal diseases, etc. (Giordano et al. 1990; Prehn and Krieglstein 1993; Ahlemeyer et al. 1999; Wesołowska et al. 2006). The efficacy of a few sesquiterpene lactones, such as damsin and coronopilin from *Ambrosia arborescens* Mill. (Asteraceae) (Villagomez et al. 2015) and arglabin from *Artemisia glabella* Kar. et Kir. (Compositae) (Zhangabylov et al. 2004), has already been proved. Studies have shown that the cytotoxicity of sesquiterpene lactones depends on the α-methylene-γ-lactone moiety, while other parts only affect the extent of cytotoxicity by their steric and chemical properties (Kupchan et al. 1971). The α-methylene-γ-lactone moiety of sesquiterpene lactones acts by alkylating the thiol groups of many transcription factors and other proteins, affecting their expression and function. Therefore, sesquiterpene lactones may inhibit different enzymes playing a key role in biological processes, such as replication, transcription, and translation. One such target of sesquiterpene lactones is the p65 subunit of nuclear factor kappa B (NFκB), which regulates many biological processes, such as cell survival, proliferation, development, angiogenesis, invasion, metastasis, and immune response. Sesquiterpene lactones alkylate Cys38 and Cys120 of the p65 subunit, modulating the action of NFκB (Vazquez-Santillan et al. 2015).

The preliminary screening of compound **1** by MTT assay for its effect on three cell lines (HeLa, MDA MB-231, and A549) showed a significant decrease (*p* < 0.05) in cell viability. Compound **1** showed strong cytotoxicity against HeLa cells (IC_50_ = 0.9 µg/mL) compared to MDA MB-231 (IC_50_ = 4 µg/mL) and A549 (IC_50_ = 3.8 µg/mL) cells, indicating that compound **1** has higher cytotoxicity against HeLa cells, so we selected it for apoptosis and DNA damage detection. The LDH release in compound **1**–treated HeLa cells was 18% and 76% at the lowest (0.5 µg/mL) and highest (15 µg/mL) concentrations, indicating disruption of the cell membrane structure, which was evident by the loss of cell attachment, round or swelled cells, and elongated cells. In addition, the population of viable cells decreased and the percentage of apoptotic cells increased (early/late apoptosis cells in 0.5 and 0.75 µg/mL were 7.58%/5.28% and 6.65%/9.23%, respectively) in compound **1**-treated HeLa cells. Apoptosis induction analysis by (AO-EB) dual staining showed that compound **1** treatment induces the highest percentage of apoptotic (20%) and necrotic (8%) cells at the highest concentration of 0.75 µg/mL. In addition, compound **1** treatment of HeLa cells significantly decreased the green fluorescence intensity, indicating mitochondrial membrane depolarization, which is an early sign of apoptosis. The genotoxic effect of compound **1** on HeLa cells was confirmed by measuring the comet tail length, which significantly increased (*p* < 0.05) in compound **1**-treated HeLa cells compared to control cells. We clearly observed DNA strand breaks and alkali-labile sites, confirming the ability of compound **1** to induce DNA damage at the single-cell level, which may lead to apoptosis.

The phenomenon of DNA damage by sesquiterpene lactones depends on their interaction with DNA via noncovalent binding to DNA or via DNA alkylation through the α-methylene-γ-lactone moiety (Chadwick et al. 2013). However, the possibility of a reaction between the α-methylene-γ-lactone moiety and the –OH or –N nucleophile of DNA is low compared to the affinity of the α-methylene-γ-lactone moiety towards the –SH group of proteins (Gates 2009). Therefore, compound **1** intercalates into the hydrophobic interior of the DNA groove. A similar mechanism of action has been observed in other sesquiterpene lactones, such as ragusinin (Grienke et al. 2018).

Sesquiterpene lactones also covalently modify other target proteins, in addition to NFκB, such as peroxisome proliferator-activated receptor-gamma (PPARγ), cystathionine β-synthase (CBS), CTTN, CSTB, and sarco/endoplasmic reticulum Ca^2+^-ATPase (SERCA) family of ATPases, UbcH5c, TrxR, and HSP72 (Lagoutte and Winssinger 2017; Shin et al. 2017). In this study, we explored the potential of compound **1** to inhibit PLK-1 using computational approaches. PLK-1 is a suitable target for many compounds isolated from natural sources (Rizvi et al. 2014; Stevenson et al. 2002). It plays a significant role in the regulation of various checkpoints during cell division (Malumbres and Barbacid 2009; Reinhardt and Yaffe 2013) and is also a hot target for cancer treatment as its overexpression or over activation often leads to uncontrolled cell growth, which, in turn, may lead to metastasis. PLK-1 overexpression is reported in many cancers, such as colorectal cancer, breast cancer, lung cancer, oesophagus and stomach cancer, pancreatic cancer, ovarian cancer, skin cancer, endometrial carcinomas, and head and neck squamous cell carcinomas (Wolf et al. 1997; Knecht et al. 1999; Tokumitsu et al. 1999; Takai et al. 2001; Kneisel et al. 2002; Takahashi et al. 2003; Gray et al. 2004; Weichert et al. 2005; Strebhardt 2010). The structural features of the PLK-1 active site have been identified by analyzing the X-ray structure of PLK-1 bound with a non-hydrolyzable ATP analog PHA-680626, which interacts with crucial residues of PLK-1 that are involved in ATP binding, such as Lys82, Glu131, and Asp194 (Kothe et al. 2007); some unique residues of PLK-1, such as Phe58 and Arg134; and some hotspot residues, such as Cys67 (located in the binding pocket rooftop), Leu132 (the hinge region residue), and Phe183 (located at the bottom of the ATP-binding pocket). A previous molecular docking report showed that PHA-680626 binds at the catalytic site and interacts with amino acid residues, such as Arg57, Phe58, Leu59, Gly60, Lys61, Gly62, Ala65, Lys66, Cys67, Ala80, Lys82, Ile83, Val114, Leu130, Glu131, Leu132, Cys133, Arg134, Arg136, Gly193, and Asp194 (AlAjmi et al. 2018).

In this study, we confirmed the cytotoxic potential of compound **1** by performing molecular docking and simulation studies with the PLK-1 kinase domain. Compound **1** binds to the PLK-1 active site and forms a stable complex. A comparison of the binding modes of compound **1** and PHA-680626 showed that residues, such as Leu59, Gly60, Lys61, Gly62, Ala65, Lys66, Cys67, Ala80, Lys82, Cys133, and Arg136, are commonly involved in the interaction with PLK-1. In addition, compound **1** interacts with some significant amino acid residues of PLK-1 that are involved in ATP binding at the catalytic site (Lys82) and hotspot residues, such as Cys67 and Phe183. The stability of compound **1** and the PLK-1 kinase domain was achieved by an MD simulation, which indicated that compound **1** forms a stable complex, making it a suitable inhibitor of PLK-1 activity.

## Conclusions

In this report, we isolated racemolactone I (a new cytotoxic sesquiterpene lactone) from the roots of *I. racemosa*. Racemolactone I demonstrated cytotoxic potential against cervical cancer (HeLa) cell lines and induced apoptosis by breaking down double-stranded DNA, leading to LDH release and mitochondrial membrane depolarization. The sesquiterpene lactones exerted their cytotoxic property by modifying the p65 subunit of NFκB. However, other protein targets cannot be ruled out which plays an important part in cell growth. The molecular docking analysis of racemolactone I exhibited that it binds and interacts with ATP-binding site of PLK-1 and key amino acid residues, respectively. In addition, MD simulation confirmed a stable compound **1**–PLK-1 complex. Our findings revealed that racemolactone I has the ability to be developed as an excellent natural product-derived PLK-1 inhibitor, and further biological studies should be conducted to confirm its use as an anticancer drug.

## Supplementary Material

Supplementary_Material_R2.docx
