# Supplementary material for: Cytotoxicity and molecular docking analysis of racemolactone I, a new sesquiterpene lactone isolated from Inula racemosa
Source: Pharm Biol. 2021 Jul 11;59(1):941–52. doi: 10.1080/13880209.2021.1946090 (PMC8274518; doi:10.1080/13880209.2021.1946090)
Supplement: Supplementary_Material_R2.docx [file IPHB_A_1946090_SM7880.docx]

**Supplementary Material**

Cytotoxicity and molecular docking analysis of racemolactone-I, a new sesquiterpene lactone isolated from *Inula racemosa*

Perwez Alam^1^*, Rama Tyagi^2#^, Mohammad Abul Farah^3^, Md. Tabish Rehman^1^, Afzal Hussain^1^, Mohamed Fahad Alajmi^1*^, Nasir Ali Siddiqui^1^, Khalid Mashai Alanazia^3^, Saima Amin^4^, Mohd. Mujeeb^5^, Showkat R. Mir^2^*

^1^Department of Pharmacognosy, College of Pharmacy, King Saud University, Riyadh- 11451, Saudi Arabia.

^2^Phyto-pharmaceutical Research Lab., School of Pharmaceutical Education and Research, Jamia Hamdard, New Delhi, India.

^3^Department of Zoology, College of Science, King Saud University, Riyadh- 11451, Saudi Arabia.

^4^Department of Pharmaceutics, School of Pharmaceutical Education and Research, Jamia Hamdard, PO Hamdard Nagar, New Delhi, India.

^5^Department of Pharmacognosy and Phytochemistry, School of Pharmaceutical Education and Research, Jamia Hamdard, New Delhi, India.

# Author to be considered as co-first author.

*****Corresponding Authors:

Perwez Alam, [aperwez@ksu.edu.sa](mailto:aperwez@ksu.edu.sa) (+966-551362901);

Mohamed F. Alajmi, [malajmii@ksu.edu.sa](mailto:malajmii@ksu.edu.sa) (+966-505151846);

Showkat R. Mir, [showkatrmir@gmail.com](mailto:showkatrmir@gmail.com) (+91-9811385772).


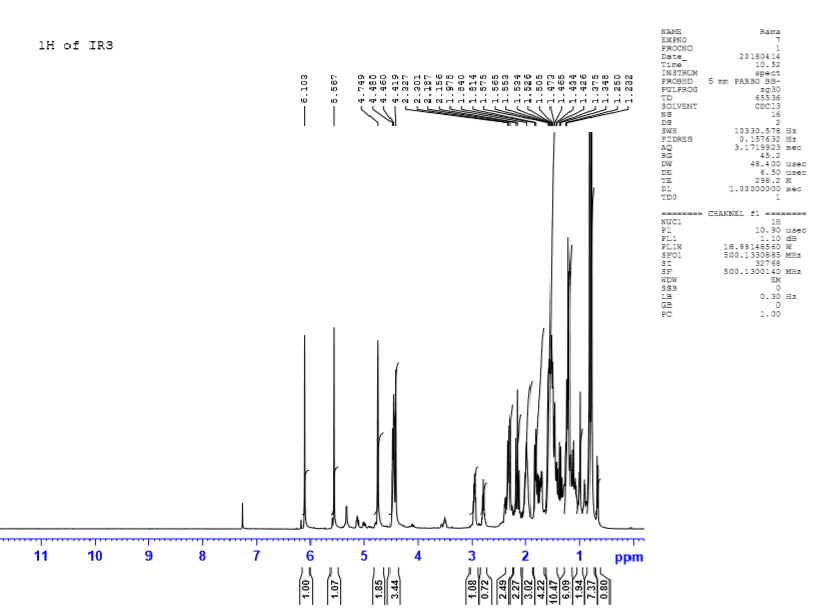


**Figure S1.** ^1^H NMR spectrum of compound 1

**Figure S2.** ^13^C NMR spectrum of compound 1

**Figure S3.** DEPT spectrum of compound 1**Figure S4.** COSY spectrum of compound 1

**Figure S5.** COSY expended spectrum of compound 1

**Figure S6.** HSQC spectrum of compound 1

**Figure S7.** HSQC expended spectrum of compound 1

**Figure S8.** HMBC spectrum of compound 1

**Figure S9.** HMBC expended spectrum of compound 1

**
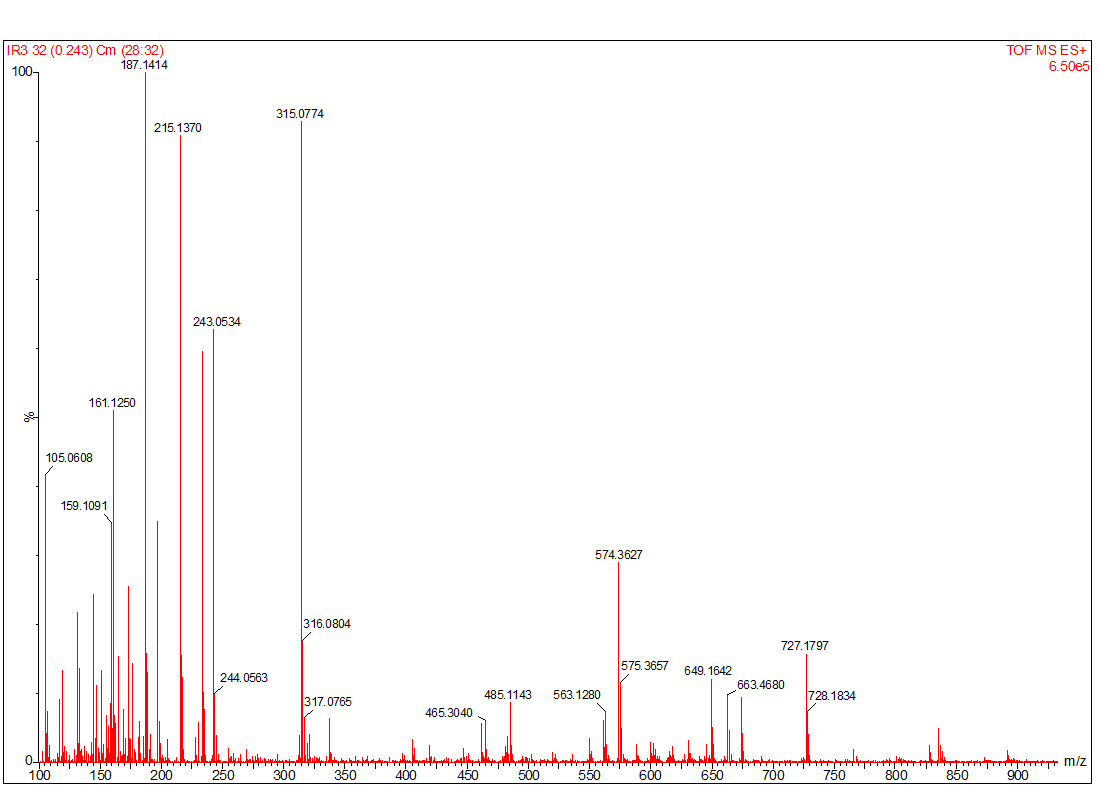
**

**Figure S10.** Mass spectrum of compound 1
